# Vertebral artery dissection associated with viral meningitis

**DOI:** 10.1186/1471-2377-12-79

**Published:** 2012-08-22

**Authors:** Xudong Pan, Aijun Ma, Kun Wang, Shumin Nie, Mei Wu

**Affiliations:** 1 Department of Neurology, The Affiliated Hospital of the Medical College of Qingdao University, 59 Haier Road, Qingdao, Shandong Province 266100, PR China; 2 Department of Ultrasonic Diagnosis, The Affiliated Hospital of the Medical College of Qingdao University, Qingdao, Shandong Province, 266003, PR China; 3 Laboratory of Human Micromorphology, The Medical College of Qingdao University, Qingdao, Shandong Province, 266071, PR China

**Keywords:** Vertebral artery dissection, Cerebral ischemia, Viral meningitis, Infection

## Abstract

**Background:**

Vertebral artery dissection (VAD) is often associated with trauma or occurs spontaneously, inevitably causing some neurological deficits. Even though acute infection can be related to the development of spontaneous VAD (sVAD), VAD associated with viral meningitis has never been reported in the literature.

**Case presentation:**

A 42-year-old man with fever, sore throat, and runny nose developed sudden onset of occipital headache, vertigo, transient confusion, diplopia, and ataxia. Brain stem encephalitis was diagnosed initially because the cerebrospinal fluid (CSF) study showed inflammatory changes. However, subsequent diffusion-weighted (DWI) magnetic resonance imaging of his brain demonstrated left lateral medullary infarction, and the digital subtraction angiography (DSA) confirmed VAD involving left V4 segment of the artery. Consequently, the patient was diagnosed as VAD accompanied by viral meningitis.

**Conclusion:**

This case suggests that viral meningitis might lead to inflammatory injury of the vertebral arterial wall, even sVAD with multiple neurological symptoms.

## Background

It is believed that vertebral artery dissection (VAD) is often associated with trauma and even less violent activities such as coughing or chiropractic manipulation [1,2]. VAD can also arise spontaneously, named spontaneous VAD (sVAD). The precise pathogenesis of sVAD is still not clear. It was reported that VADs might be associated with acute infection [3,4]. Nevertheless, sVAD associated with viral meningitis has not been reported yet up to now.

VADs may present with a variety of neurological symptoms, and the most frequent presentation is unilateral neck pain or occipital headache, with or without signs of cerebellar or medullary infarction. We report here a case of sVAD associated with viral meningitis which was secondary to the upper respiratory tract infection. This patient manifested with variable symptoms including fever, headache, diplopia, vertigo, confusion, and ataxia. Leukocytosis and elevated protein level were found via cerebrospinal fluid (CSF) study, infarction of left lateral medulla oblongata was demonstrated via DWI-MRI study, and finally left sVAD was confirmed by digital subtraction angiography (DSA).

## Case presentation

A 42-year old man suffered from fever, sore throat, mild bilateral headache, and runny nose for 5 days. An acute onset of occipital headache and vertigo occurred one day before admission. He had transient diplopia and confusion for several hours on the day of admission, and received intravenous drip of penicillin. Shortly after the relief of confusion, he experienced dysphagia, hoarseness, as well as ataxia of the left upper and lower extremities. He had no coughing or vomiting during this period. The patient was healthy previously, and denied any head or neck trauma and chiropractic manipulation. He smoked 10 to 15 cigarettes daily, without any use of alcohol or recreational drugs.

On physical examination, his temperature was 37.6°C, pulse 72 beats/min, blood pressure 130/80 mmHg. No remarkable positive signs were detected by the lung, cardiac, and abdominal examinations. Neurologic examination revealed that the pupils were equal and reactive to light, but horizontal nystagmus, hoarse voice and decreased movement of the left soft palate were noticed. He had normal strength in all limbs but decreased pain sensation on the left face and right limbs. In addition, he had left-sided finger-to-nose and heel-knee-shin dysmetria. Neck stiffness was mild, but Kernig's and Brudzinski's signs were not observed.

Admission laboratory studies showed normal serum electrolyte and metabolic panel. The blood count showed 13,760 white blood cells (WBCs) per cubic millimeter with 91% polymorphonuclear leukocytes (PMN). Coagulation tests and erythrocyte sedimentation rate were normal. Venereal Disease Research Laboratory (VDRL) and human immunodeficiency virus (HIV) antibody test were negative. High sensitive C-reactive protein (hs-CRP) (3.72 mg/dL) and fibrinogen (4.1 g/L) were elevated. Connective tissue disease work-up was negative. He had normal chest X-ray and electrocardiography. Lumbar puncture on admission showed an opening pressure of 190 mmH_2_O. It was a traumatic tap, so three serial samples of fluid were taken. Being clear and colorless, the third one was analyzed. The CSF study showed 84 WBCs per cubic millimeter with 90% lymphomonocyte and 10% PMN, 236 red blood cells per cubic millimeter, normal glucose concentration (3.3 mmol/L), increased protein concentration (744 mg/L), increased IgA and IgG concentration (11.0 mg/L and 38.3 mg/L respectively), and normal IgM level (0.23 mg/L). The IgG index was 78%. CSF cytology, smear for acid-fast bacilli, Gram stain and Indian ink were all negative. Viral immunological reactions were negative (herpes simplex virus I and II, Epstein Barr virus and cytomegalovirus), so were CSF polymerase chain reactions for enterovirus, herpes simplex virus I and II.

**Figure 1:**
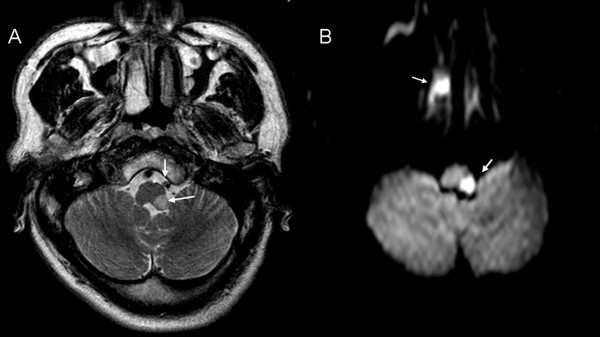
**MRI of the patient: (A) Axial T**_**2**_**-weighted image showing hyperintense signal in the left lateral medullary and narrow lumen of the left vertebral artery; (B) Diffusion-weighted image showing hyperintense signal in the left lateral medullary and hyperintense signal of left inferior turbinate.**

**Figure 2:**
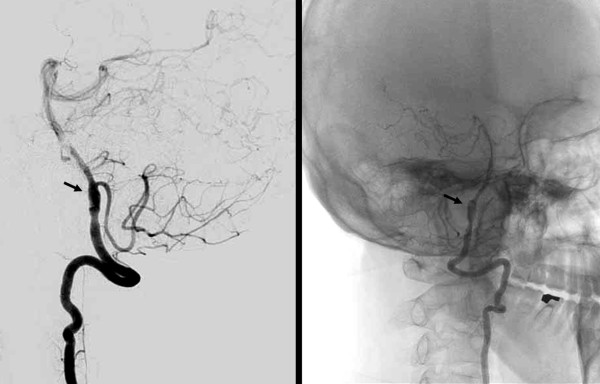
DSA of the patient: Left vertebral angiograms showing focal luminal dilatation involving left V4 segment of the artery (arrow) just proximal to PICA and insufficient blood flow at the distal portion.

CT of the head and electroencephalography were normal. T2-weighted MRI of the brain on the second day showed hyperintense signal in the left lateral medulla and narrowing of the lumen of the left vertebral artery. DWI images also showed hyperintense signal in the left lateral medulla and hyperintense signal of left inferior turbinate, suggesting medullary infarction and rhinitis (Figure1). DSA showed focal luminal dilatation involving left V4 segment of the artery just proximal to posterior inferior cerebellar artery (PICA) and insufficient blood flow to its distal portion. Dissection of the left vertebral artery at the level of V4 segment was confirmed (Figure2).

Intravenous heparin was administered to the patient. After 24 hours of treatment, his vertigo was relieved. Ataxia and headache alleviated by day 4. He was discharged 10 days later after admission with only symptom of dysphagia. At one-month follow-up, the patient recovered completely, and repeated CSF study was normal.

## Discussion

With the symptoms of fever, headache, confusion, diplopia, and signs of neck stiffness, intracranial infection was firstly considered. Furthermore, increased intracranial pressure, normal glucose, monocytic pleocytosis, increased protein concentration, increased IgA and IgG concentration of CSF suggested viral inflammatory reaction although the immunological reactions and polymerase chain reactions for some common viruses were negative. Consequently, our case was initially diagnosed as brainstem encephalitis. However, resolution of confusion and diplopia in several hours, and the findings of left lateral medullary infarction on the DWI MRI indicated otherwise. Subsequent DSA suggested dissection of the left vertebral artery involving V4 segment proximal to PICA. As the patient had fever, headache, neck stiffness and inflammatory CSF, the diagnosis of VAD accompanied by viral meningitis was preferred.

VAD was once considered to be a rare event, but recently it has been recognized as an important cause of ischemic stroke in middle-aged adults (10–25% of these cases) [1]. About 60% of VAD patients have symptoms of vertebrobasilar circulation ischemia, such as vertigo, hoarseness, dysphagia and cerebellar dysfunction [5]. Occipital headache and neck pain are common symptoms and occur in 70% of patients with VAD [6]. The acute onset of occipital headache in our patient was thought to result from VAD. The normal MRI signs of the midbrain and pons suggested that the transient confusion and diplopia were due to ischemia rather than brainstem encephalitis. The development of intramural hematoma due to the dissection resulted in narrow lumen and hypoperfusion of vertebrobasilar system. With recanalization or reperfusion, the patient’s consciousness recovered rapidly. Since ipsilateral PICA was usually not well compensated, infarction of left lateral medullary developed, and the patient experienced dysphagia, ataxia and numbness of left face and right extremities. The other possible mechanism might be related to transient embolization of vertebrobasilar system from the VAD, or related to a combination of embolization and local occlusion [7].

The pathogenesis of VADs is actually not completely understood now. In the past, it was believed that mild or severe neck trauma caused mechanical stretching and compression of the vessel resulting in intimal disruption and intramural hemorrhage [5]. However, mechanical stress tends to affect extracranial segments, including V1 to V3, because of its mobility and prone to be damaged by bony structures [7]. Recent histopathological studies of both internal carotid artery dissection (ICAD) and VAD revealed that there was no mechanical damage being observed [8,9]. In our case, only the intracranial segement of the vertebral artery was involved, and the patient had no history of trauma, even a possible damage from coughing or sneeze. We therefore believe that the patient’s VAD was associated with viral meningitis. There was an inflammation-associated injury of the arterial wall that led to weakness of vessel wall and subsequent dissection. The leucocytosis and elevated hs-CRP were indicative of this proposed inflammatory process. Furthermore, significantly elevated WBCs and protein level in CSF confirmed the inflammatory reaction of central nervous system.

Recent observations showed that infections may play an important role in both ICAD and VAD [3,4,10]. Microbial agents themselves may cause substantial damage to the vascular wall, as destructive changes after infection have been shown to be centered in the tunica media where dissections occur [11-13]. Different viruses and bacteria were suspected as being the responsible agents but previous studies failed to prove a common microbe [1,14,15]. Although some authors attributed the association between infection and dissection to mechanical stress (e.g., coughing, sneezing and vomiting) [11], the multivariate analysis in Grau’s study confirmed that the dissection was independently associated with a diagnosis of recent infection rather than mechanical factors occurring during the infection [10]. Latest data showed persistently elevated hs-CRP values in ICAD and VAD patients, and elevated WBCs and CRP in spontaneous dissections as compared to traumatic dissections [16,17]. These findings may indicate that the inflammatory response is more likely responsible for dissections [15].

## Conclusion

Our case suggested that viral meningitis might lead to inflammatory injury of the vertebral arterial wall that subsequently developed sVAD with multiple neurological symptoms. However, VAD is probably the consequence of a complex vasculopathy process that may be related to various environmental factors and inborn predispositions. Therefore, further investigations with larger patient groups and of the sensitivity and specificity of inflammatory markers, as well as histopathological data of dissected vessels, are needed to support our hypothesis.

## Consent

Written informed consent was obtained from the patient for publication of this case report and any accompanying images. A copy of the written consent is available for review by the Editor-in-Chief of this journal.

## Abbreviations

VAD: Vertebral artery dissection; sVAD: Spontaneous VAD; CSF: Cerebrospinal fluid; MRI: Magnetic resonance imaging; DWI: Diffusion-weighted; DSA: Digital subtraction angiography; PMN: Polymorphonuclear leukocytes; hs-CRP: High sensitive C-reactive protein; CT: Computed tomography; PICA: Posterior inferior cerebellar artery; ICAD, Internal carotid artery dissection.

## Competing interests

All authors declare no competing interest.

## Authors’ contributions

XP and AM contributed to the conception and design. XP, AM and KW took care of collecting the clinical information. AM, KW carried out literature search. AM, XP, SN and MW drafted and revised the manuscript. All authors have read and approved the final manuscript.

## Pre-publication history

The pre-publication history for this paper can be accessed here:

http://www.biomedcentral.com/1471-2377/12/79/prepub
